# In utero fetal left ventricular rupture and pseudoaneurysm formation: a case report

**DOI:** 10.1186/s12884-021-03869-4

**Published:** 2021-05-20

**Authors:** Sarah Heland, Sarah Hope, Andrew Edwards, Rebecca Chalmers, Alice Stewart, Annie Kroushev, Bennett Sheridan, Stuart Hooper, Kirsten R. Palmer

**Affiliations:** 1 Department of Obstetrics and Gynaecology, Monash Health, Clayton, VIC Australia; 2 Department of Paediatric Cardiology, Monash Heart, and Monash Cardiovascular Research Centre, Clayton, Australia; 3 Department of Obstetrics and Gynaecology, Peninsula Health, and The Ritchie Centre, Hudson Institute of Medical Research, Clayton, Australia; 4 Monash Newborn, Monash Health, Clayton, Australia; 5 Cardiac Intensive Care, Royal Children’s Hospital, Parkville, Australia; 6 The Ritchie Centre, Hudson Institute of Medical Research, Clayton, Australia; 7 Department of Obstetrics and Gynaecology, Monash Health, and The Ritchie Centre, Hudson Institute of Medical Research, Clayton, Australia

**Keywords:** Cardiac pseudoaneurysm, Ventricular rupture, Interventional drainage, Perinatal cardiology

## Abstract

**Background:**

Cardiac ventricular aneurysms affect 1 in 200,000 live births. To the best of our knowledge, no reported cases of a left ventricular pseudoaneurym and in utero rupture exist to guide optimal management.

**Case presentation:**

We present a case of fetal left ventricular rupture with a large pericardial effusion, cardiac tamponade and subsequent pseudoaneurysm formation with concerns for a poor prognosis. Interventional drainage of the pericardial effusion led to resolution of tamponade and significant improvement in fetal condition. A multidisciplinary team was utilised to plan birth to minimise risk of pseudoaneurysmal rupture and a catastrophic bleed at birth.

**Conclusion:**

For similar cases we recommend consideration of birth by caesarean section, delayed cord clamping and a prostaglandin E1 infusion, to reduce the systemic pressures on the left ventricle during transition from fetal to neonatal circulations, until definitive surgical repair. In this case, this resulted in a successful outcome.

## Background

Cardiac ventricular aneurysms affect 1 in 200,000 live births [1]. The first reported case diagnosed prenatally was published in 1990, however, to the best of our knowledge, no reported cases of a left ventricular pseudoaneurym and in utero rupture exist to guide optimal management [2].

## Case presentation

**Fig. 1:**
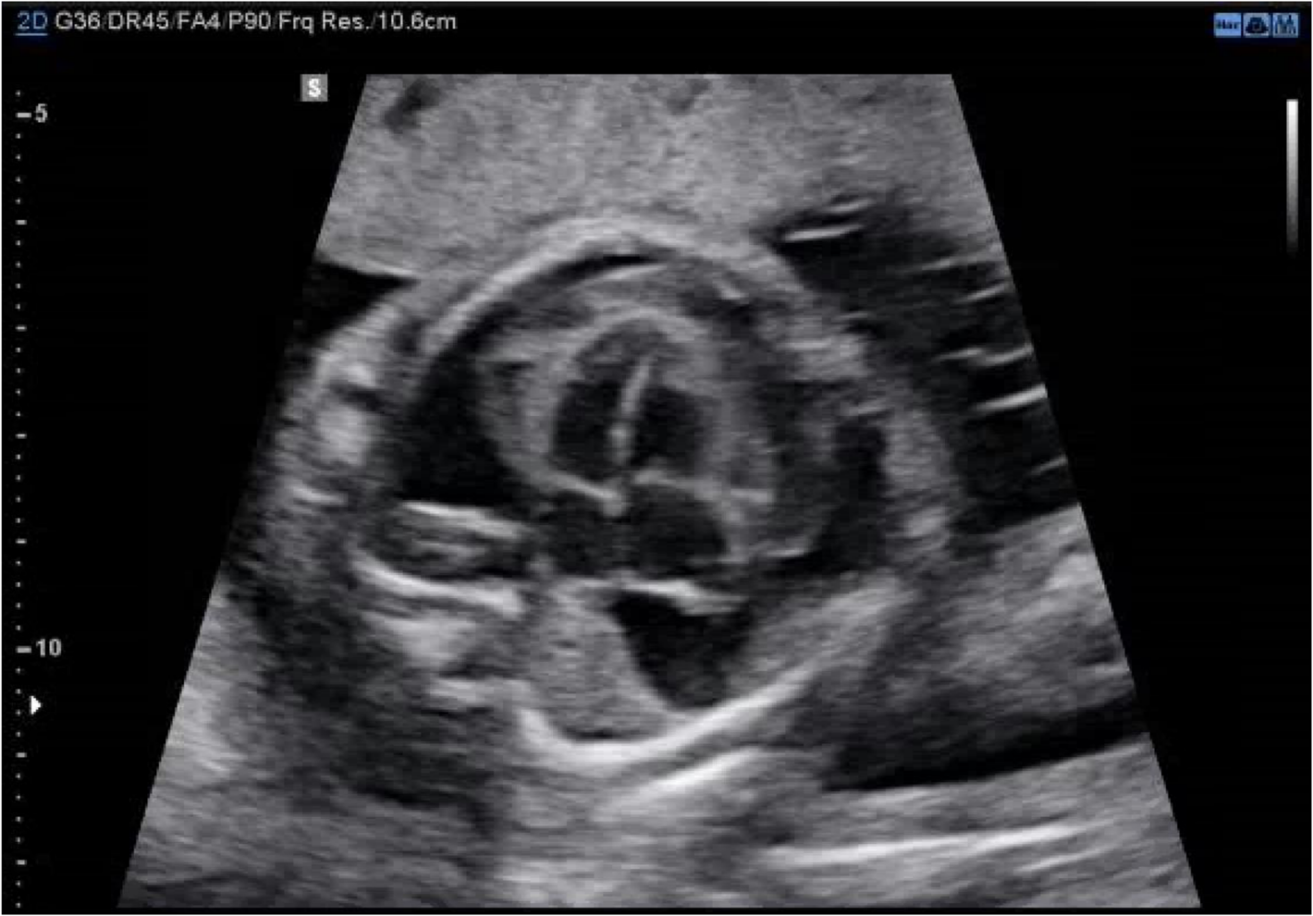
Axial 4-chamber view of the fetal heart at 23 + 1 weeks’ gestation with a complex epicardial mass and a massive pericardial effusion seen

A 25-year-old primiparous woman was referred for tertiary specialist fetal assessment at 23 weeks’ gestation following a mid-trimester ultrasound indicating right outflow tract obstruction with pericardial effusion. Her pregnancy was previously uncomplicated. She had no significant past history. On review, a complex epicardial mass associated predominately with the left ventricle and a massive pericardial effusion was seen (Fig. 1), suggestive of an epicardial bleed. Remaining fetal anatomy was normal with no evidence of fetal hydrops or anaemia. An uncomplicated amniocentesis was performed and returned a normal Fluorescence In Situ Hybridization (FISH) and karyotype result; maternal infection screening was negative.

At 24 + 1 weeks’ gestation, the fetal pericardial effusion appeared stable but with an increasing epicardial thrombus. There was bidirectional flow between a pseudoaneurysmal defect and the left ventricle. An abnormal ductus venosus Doppler waveform, indicating poor ventricular function secondary to cardiac tamponade, and new onset ascites was evident.

**Fig. 2:**
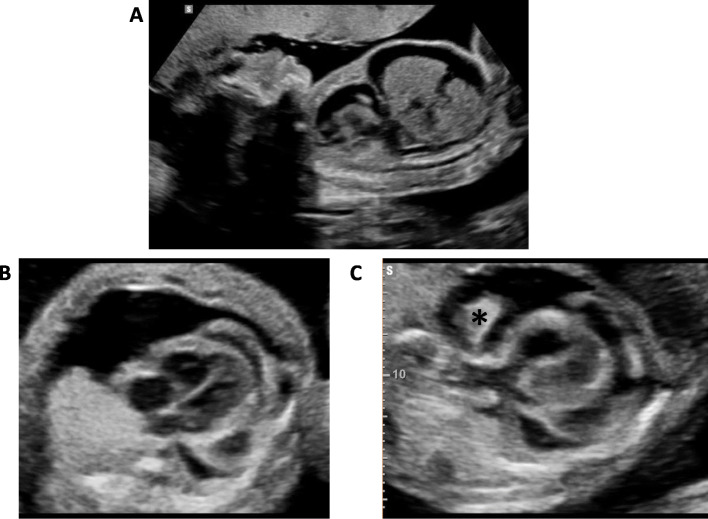
Ultrasound assessment at 25 weeks’ gestation, with evidence of fetal hydrops with ascites and a pleural effusion seen on sagittal view (**a**), a 4-chamber axial view of the heart shows persisting complex epicardial mass predominately associated with the left ventricle (**b**) and features suggestive of pseudoaneurysmal rupture with heterogenous material consistent with blood clot (*) seen in the pericardial effusion (**c**)

Drainage of the massive pericardial effusion was deferred at 25 weeks’ gestation, despite worsening hydropic features, due to bleeding into the pericardial space raising concerns for pseudoaneurysmal rupture (Fig. 2). At 26 + 1 weeks’ a pericardial drainage of 40 ml of haemolysed blood-stained fluid was performed. Weekly follow up showed a small, gradually reducing, pericardial effusion, with resolution of the ascites and normalisation of the ductus venosus Doppler waveform.

A multidisciplinary team meeting with input from maternal fetal medicine, neonatology, paediatric cardiology, paediatric cardiac intensive care and perinatal physiology was conducted at 31 weeks’ gestation to plan birth and postpartum care. Concerns existed of pseudoaneurysmal rupture and a potential catastrophic bleed during transition, as afterload pressures on the pseudoaneurysm increase due to increased systemic vascular resistance upon cord clamping and the loss of the low resistance placental circulation.

A caesarean birth was advised due to concerns of fetal stress and transient increases in placental resistance during labour increasing left ventricular afterload and the risk of pseudoaneurysmal rupture. Birth at term to minimise risks of prematurity and optimise birthweight prior to definitive surgical repair on cardiac bypass soon after birth was planned [3].

**Fig. 3:**
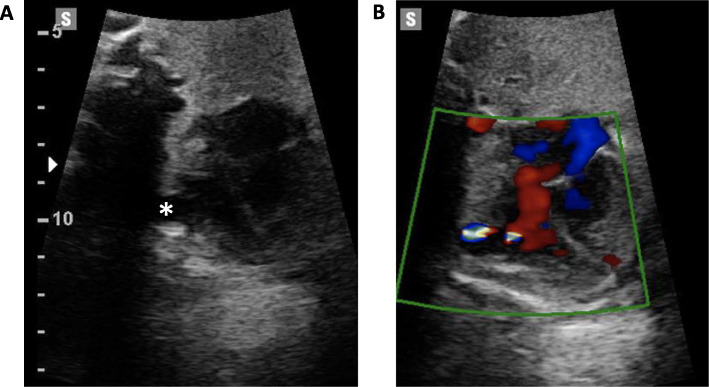
Axial 4-chamber view of the fetal heart at 35 + 1 weeks’ gestation with a pseudoaneurysmal sac evident (*) arising from the left ventricle and resolution of the pericardial effusion (**a**). Blood flow into the pseudoaneurysm was clearly seen (**b**)

A fetal MRI showed normal neuroanatomy at 33 weeks’ gestation. At 35 + 1 weeks’, a large left ventricular pseudoaneurysm persisted, but the pericardial effusion had completely resolved (Fig. 3).

An elective caesarean birth occurred at 37 + 5 weeks’ gestation following steroids to minimise the need for respiratory support. Cord clamping was deferred until after respiration was established to reduce pulmonary vascular resistance and allow left-to-right shunting through the ductus arteriosus to minimise the increase in left ventricular afterload associated with cord clamping. A female infant was born weighing 3209 g with Apgars of 9 at 1 min and 9 at 5 min. In the immediate post-partum period, a prostaglandin E1 infusion was commenced to maintain ductal patency and reduce systemic and pulmonary vascular resistance. She remained stable and was transferred on day 1 of age to a quaternary paediatric cardiac centre for surgical management, undergoing aneurysmal resection with patch closure and ligation of a patent ductus arteriosus at 10 days of age. She made a good post-operative recovery and was discharged home at 19 days of age. On follow-up at 4 months of age she is thriving with a repeat echocardiogram showing good biventricular function.

## Discussion and conclusions

Here we describe a case of fetal cardiac left ventricular pseudoaneurysm with in utero rupture that resulted in a successful outcome. Due to the small isolated defect, it is thought to have occurred secondary to a single coronary artery occlusion causing a localised myocardial infarction. Counselling the parents on the likelihood of having a live born infant and the subsequent prognosis following birth was challenging due to the rarity of the condition.

In view of the maternal primigravity and age, vaginal birth was considered to minimise maternal surgical morbidity in light of uncertain neonatal outcome. As labour and vaginal birth are known to cause transient episodes of fetal and placental hypoxia, leading to increased fetal acidosis and systemic arterial pressures, this mode of birth was thought to increase the possibility of pseudoaneurysmal rupture [4]. Utilising joint decision making with the parents, a caesarean birth was chosen as the optimal mode of delivery to minimise fetal risk.

Many key cardiovascular changes occur during transition from a fetal to neonatal circulation, including a significant reduction in the pulmonary vascular resistance, an increase in systemic vascular resistance, and closure of the ductus arteriosus [5]. Prior to birth, left ventricular preload is predominately derived from umbilical venous return, while after birth it is from the pulmonary circulation [6]. Clamping of the umbilical cord removes the low resistance placental bed which significantly increases systemic vascular resistance and arterial blood pressure, such that in the first hour following birth the left ventricular cardiac output doubles [5, 6]. As the left ventricle transitions from pumping against the low resistance placental circulation to the high resistance neonatal systemic circulation, there is a significant increase in afterload, and in this case, an increased risk of pseudoaneurysmal rupture and catastrophic bleed [6]. To minimise this risk, umbilical cord clamping was delayed until neonatal respiration was well established, so that the pulmonary circulation could act as an alternate lower resistance pathway for left ventricular output, due to left-to-right ductal shunting. This prevents the rapid and large increase in left ventricular afterload associated with cord clamping [7]. The neonate was commenced on a prostaglandin E1 infusion to maintain a patent ductus arteriosus, minimizing systemic vascular resistance and decreasing left ventricular pressures [8].

We would recommend that in similar cases caesarean birth, deferred cord clamping and immediate commencement of a prostaglandin E1 infusion be considered until definitive surgical repair. Importantly, the possibility of an excellent outcome is also valuable in counselling and guiding families that may be impacted by this rare complication in the future.

## Data Availability

Data sharing is not applicable to this article as no datasets were generated or analysed during the current study.

## Abbreviations

FISH: Fluorescence In Situ Hybridization
MRI: Magnetic Resonance Imaging
